# Deletion of the Mouse Homolog of *CACNA1C* Disrupts Discrete Forms of Hippocampal-Dependent Memory and Neurogenesis within the Dentate Gyrus

**DOI:** 10.1523/ENEURO.0118-16.2016

**Published:** 2016-11-28

**Authors:** Stephanie J. Temme, Ryan Z. Bell, Grace L. Fisher, Geoffrey G. Murphy

**Affiliations:** 1 Neuroscience Graduate Program, University of Michigan, Ann Arbor, Michigan 48109-0069; 2 Molecular & Behavioral Neuroscience Institute, University of Michigan, Ann Arbor, Michigan 48109-0069; 3 Department of Molecular & Integrative Physiology, School of Medicine, University of Michigan, Ann Arbor, Michigan 48109-0069

**Keywords:** L-type voltage-gated calcium channel, pattern completion, pattern separation

## Abstract

L-type voltage-gated calcium channels (LVGCCs) have been implicated in various forms of learning, memory, and synaptic plasticity. Within the hippocampus, the LVGCC subtype, Ca_V_1.2 is prominently expressed throughout the dentate gyrus. Despite the apparent high levels of Ca_V_1.2 expression in the dentate gyrus, the role of Ca_V_1.2 in hippocampal- and dentate gyrus-associated forms of learning remain unknown. To address this question, we examined alternate forms of hippocampal-dependent associative and spatial memory in mice lacking the mouse ortholog of *CACNA1C* (*Cacna1c*), which encodes Ca_V_1.2, with dentate gyrus function implicated in difficult forms of each task. We found that while the deletion of Ca_V_1.2 did not impair the acquisition of fear of a conditioned context, mice lacking Ca_V_1.2 exhibited deficits in the ability to discriminate between two contexts, one in which the mice were conditioned and one in which they were not. Similarly, Ca_V_1.2 knock-out mice exhibited normal acquisition and recall of the location of the hidden platform in a standard Morris water maze, but were unable to form a memory of the platform location when the task was made more difficult by restricting the number of available spatial cues. Within the dentate gyrus, pan-neuronal deletion of Ca_V_1.2 resulted in decreased cell proliferation and the numbers of doublecortin-positive adult-born neurons, implicating Ca_V_1.2 in adult neurogenesis. These results suggest that Ca_V_1.2 is important for dentate gyrus-associated tasks and may mediate these forms of learning via a role in adult neurogenesis and cell proliferation within the dentate gyrus.

## Significance Statement

Recent genome-wide association studies have implicated the gene *CANA1C*, which encodes the L-type voltage-gated calcium channel Ca_V_1.2 as a risk factor for psychiatric disease. Here we examine mice lacking the mouse ortholog of *CANA1C*. We find that, while seemingly normal, these mice lack the ability to successfully learn tasks that require the discrimination of environmental cues or in which the cues are limited. This type of learning, often referred to as pattern separation/completion, is thought to require the birth and survival of neurons in the dentate gyrus subregion of the hippocampus. Interestingly, mice lacking Ca_V_1.2 exhibit reduced neurogenesis in this brain region. Our results suggest an intriguing link among a psychiatric risk allele, neurogenesis, and pattern separation/completion.

## Introduction

In neurons, activity-dependent increases in intracellular calcium are mediated in large part by calcium influx through L-type voltage-gated calcium channels (LVGCCs). As a class of channels, the LVGCCs have been implicated in a wide range of neurophysiological functions, including the regulation of intrinsic neuronal excitability (Kaczorowski, 2011), synaptic plasticity (Kapur et al., 1998; Zakharenko et al., 2001), and transcriptional activation (Deisseroth et al., 1998), as well as cognition (Bauer et al., 2002; Cain et al., 2002; Davis and Bauer, 2012).

Of the four major LVGCC subtypes, Ca_V_1.2 and Ca_V_1.3 are abundantly expressed within the mammalian brain. However, differential expression patterns (Hell et al., 1993) and biophysical characteristics (Lipscombe et al., 2004) suggest that Ca_V_1.2 and Ca_V_1.3 may have distinct roles in neuronal function and behavior. In the hippocampus, Ca_V_1.3 is expressed in the soma and proximal dendrites throughout the hippocampus, while Ca_V_1.2 is broadly expressed throughout CA3 and the dentate gyrus, with limited expression elsewhere (Hell et al., 1993; Marschallinger et al., 2015). *In vitro* studies of neuronal development have implicated LVGCCs in cell proliferation and neurogenesis (D'Ascenzo et al., 2006; Piacentini et al., 2008; Brustein et al., 2013). Additionally, Ca_V_1.2 has also been tied to the survival of adult-born neurons in the dentate gyrus *in vivo* (Lee et al., 2016). Previously, it has been demonstrated that Ca_V_1.3 is required for the consolidation of contextual fear (McKinney and Murphy, 2006), and a recent report (Marschallinger et al., 2015) suggests that Ca_V_1.3 is required for object location discrimination. Conversely, individual investigations of Ca_V_1.2 have yielded mixed results. Deletion of the gene *Cacna1c* (the mouse ortholog of the *CACNA1C* gene), which encodes Ca_V_1.2 did not affect contextual fear learning (McKinney et al., 2008; Langwieser et al., 2010) but did result in remote spatial learning deficits (White et al., 2008). Additionally, deficits in the visible platform discrimination water maze and the labyrinth maze were observed in a Ca_V_1.2 conditional knock-out mouse (Moosmang et al., 2005). In light of these behavioral results and the differential expression of Ca_V_1.2 in the hippocampus, we hypothesized that Ca_V_1.2 might be important in hippocampal-dependent learning in a uniquely task- and subregion-dependent manner. Unlike other regions of the hippocampus, the dentate gyrus is the location of continual cell proliferation into adulthood (Altman and Das, 1965; Gage, 2002). Adult born neurons within the dentate gyrus have been linked to unique forms of hippocampal-dependent learning (Gould et al., 1999; Shors et al., 2002; Winocur et al. 2006). While lesion studies have demonstrated a role of the hippocampus in proper contextual fear conditioning (Logue et al., 1997; Maren et al., 1997) and the standard water maze (Logue et al., 1997), disruptions in neurogenesis within the dentate gyrus do not impair the ability of animals to acquire these tasks (Shors et al., 2002; Jaholkowski et al., 2009). In contrast, more difficult learning tasks appear to rely on the dentate gyrus and are impaired when neurogenesis is decreased (Shors et al., 2002).To evaluate the role of Ca_V_1.2 in difficult versus simple learning tasks, we used Ca_V_1.2 conditional knock-out (Ca_V_1.2^cKO^) mice in which Cre was driven throughout neuronal populations (Zhu et al., 2001; Cui et al., 2008). These mice were examined for deficits in simple and complex versions of spatial and contextual learning tasks. We found that Ca_V_1.2^cKO^ mice learned normally in simple tasks, such as context fear conditioning and the standard Morris water maze, but exhibited significant deficits in complex tasks, including context discrimination and the limited cues water maze. Additionally, Ca_V_1.2^cKO^ mice were found to have a decrease in cell proliferation and decreased numbers of immature neurons in the dentate gyrus. Several genome-wide association studies have linked *CACNA1C* to a wide variety of psychiatric disorders (Sklar et al., 2008), raising the intriguing possibility that disruptions of Ca_V_1.2 function or expression may play a significant role not only in cognition, but also in neuropsychiatric disorders via modulation adult neurogenesis and dentate gyrus function.

## Materials and Methods

### Mice

All studies were conducted using naive mice. Mice were 3–7 months of age at the time of each behavioral experiment and 4 months of age at the time of tissue collection. Approximately equal numbers of males and females were used per genotype. Due to the absence of sex differences, all data are presented as an average of both male and female mice. Mice in each line were housed by sex and in groups of three to five. Throughout the course of all experiments, the investigator remained blind to the genotype of the mice. Mice were maintained in microisolation cages with a 14/10 h light/dark cycle, an average ambient temperature of 22°C, and *ad libitum* access to food and water. All experiments were conducted according to the National Institutes of Health guidelines for animal care and were performed in accordance with the University of Michigan Institutional Animal Care and Use Committee regulations. Conditional knock-out mice with neuron-specific deletion of Ca_V_1.2 (Ca_V_1.2^cKO^) and their wild-type littermates were used. Mice used in this study were on a C57BL/6:129SvEv F2 genetic background. Mice with a floxed Ca_V_1.2 exon 2 allele (Ca_V_1.2^f/+^ or Ca_V_1.2^f/f^) and maintained on a 129SvEv genetic background were first bred to transgenic mice expressing the Cre recombinase regulated by the synapsin1 promoter (Syn1-Cre^Cre/+^) and were maintained on a C57BL/6 background, producing an F1 cross. Using nonlittermate offspring from the F1 cross, heterozygous-floxed, Cre-positive mice (Ca_V_1.2^f/+^ Syn1-Cre^Cre/+^) were then crossed with heterozygous-floxed, Cre-negative mice (Ca_V_1.2^f/+^ Syn1-Cre^+/+^) to produce homozygous-floxed, Cre-positive (Ca_V_1.2^f/f^ Syn1-Cre^Cre/+^) conditional knock-out mice as well as mice categorized as wild-type or control. Mice were considered to be wild type if they were Cre negative and lacked the floxed alleles (Ca_V_1.2 ^+/+^ Syn1-Cre^+/+^). Mice were considered to be a control if they were homozygous or heterozygous for the floxed allele and Cre negative (Ca_V_1.2 ^f/f^ Syn1-Cre^+/+^; Ca_V_1.2^f/+^ Syn1-Cre^+/+^), or if they were Cre positive but lacked the floxed alleles (Ca_V_1.2^+/+^ Syn1-Cre^Cre/+^). Because no significant differences were detected between the various control groups and true wild-type mice, these groups were collapsed into a single group, which we refer to simply as “wild-type” throughout the rest of the article.

### Pavlovian fear conditioning

Fear-conditioning experiments were conducted as previously described (Temme et al., 2014). Fear-conditioning chambers were composed of clear acrylic backs and doors, aluminum sides, stainless steel grid floors with one-eighth inch spaces, and stainless steel drop pans (Med Associates). Throughout experimentation, chambers and floor pans were cleaned with 70% ethanol. Chambers were illuminated using room lights set at 150 W. Footshocks were administered through the grid via solid-state shock scramblers and electronic constant-current shock sources. Shocks were controlled by a desktop PC running Actimetrics FreezeFrame software. The behavior of each mouse was recorded and digitized using individual cameras mounted above each chamber using the Actimetrics FreezeFrame software. Mice were fear conditioned to a context using one training session per day for 2 d. Each fear-conditioning session consisted of 3 min of context exposure to the training chamber followed by three unsignaled footshocks (0.5 mA, 2 s) with 30 s between footshocks. Mice were removed from the training chamber 30 s after the last footshock. Twenty-four hours after the last training session, mice were tested for fear of the trained context using 5 min of context exposure.

### Context discrimination

During context discrimination, mice were trained to discriminate between two contexts through exposure to both contexts each day for 10 d, separated by a minimum of 6 h. One context was termed context A, and consisted of the fear-conditioning chamber as described above, room lights at 150 W, a scent of 70% ethanol, and white noise. The second context was termed context B, and was composed of red room lights, a scent of 2% acetic acid, no white noise, and the floor in the fear-conditioning chamber was covered with a speckled rubber floor covering. In context A, mice were trained each day using 3 min of context exposure followed by one unsignaled footshock (2 s, 0.5 mA). Mice were removed from the conditioning chambers 30 s after the footshock. In context B, mice received context exposure for 3 min and 32 s, which is comparable to the time spent in context A with no unsignaled footshock. The order of exposure to contexts A and B was alternated each day. Mice were tested for their fear of contexts A and B on day 10 using an exposure of 3 min and 30 s to each context in the absence of a footshock.

### Standard Morris water maze

The water maze was composed of a round white acrylic pool that was 1.2 m in diameter. Throughout experimentation, the pool was filled with water that was made opaque using nontoxic white tempera paint and heated to 27°C. A round platform made of clear acrylic and measuring 10 cm in diameter was submerged just below the surface of the water. Mice were tracked in two-dimensional space within the water maze using a digital camera mounted above the pool. Digital tracking and off-line analysis were accomplished with Actimetrics Water Maze 3 software. Mice were allowed to find the hidden platform using two back-to-back training sessions per day for 9 d. For each session, mice were released pseudorandomly into the maze facing the outside of the maze, and the time it took them to find the platform was recorded. For all sessions, mice were given 60 s to find the platform, after which point mice would be guided to the platform. Each day, mice were individually placed on the platform for 15 s before the start of session 1 and session 2. After the completion of each training session, mice were allowed to remain on the platform for a period of 15 s before being placed back in their home cage. Mice were tested for their memory of the platform location using a probe test 24 h after the last day of training. During the probe test the platform is removed and the amount of time mice spend within a specific vicinity of the platform is measured. The probe test was performed by releasing each mouse individually into the pool at a point opposite to the original platform location. Mice were then allowed to explore the maze for a period of 60 s before being removed near the original platform location.

### Limited cues water maze

During the limited cues water maze, a blue plastic barrier was used to encircle the entire water maze, blocking all visual cues in the room except for four discreet visual cues that were mounted evenly around the maze. Mice were trained to the limited cues water maze using two sessions per day for 12 d. Aside from the increase in number of training days, training was conducted in the same manner as in the standard Morris water maze. Mice were tested for their memory of the platform location using the following two probe tests: one at the beginning of day 9 of experimentation, and one 24 h after the last day of training. Probe tests were conducted as described for the standard Morris water maze.

### Bromodeoxyuridine labeling

Mice were administered bromodeoxyuridine (BrdU; 100 mg/kg) dissolved in sterile PBS, via intraperitoneal injections, once a day for 5 d in order to assess cell birth rates in the dentate gyrus. All BrdU solutions for injection were made fresh daily at a concentration of 10 mg/mL and a pH of ∼7.4, with the addition of NaOH if necessary. For each injection, mice were anesthetized using isoflurane prior to each BrdU injection and monitored until they recovered. Approximately 24 h after the last injection, mice were perfused using 0.9% sodium chloride followed by ice-cold 4% paraformaldehyde in PBS. Brains were then removed and postfixed overnight in 4% paraformaldehyde, after which time they were transferred to 30% sucrose for a minimum of 48 h or until saturation of the brain in sucrose allowed the brains to sink in solution. The brains were then frozen on dry ice, and coronal slices were made at 40 μm and stored, free floating, in cryoprotectant buffer at −20°C for later use. Brains sections were labeled for BrdU expression using immunofluorescent histochemistry. Selected tissue sections were rinsed of cryoprotectant buffer using an overnight wash in Tris, pH 7.4. Sections were then washed using Tris-buffered saline (TBS), pH 7.4, and incubated in 2 m HCl for 30 min at 32°C to denature the DNA. Sections were then neutralized using 0.1 m sodium borate for 10 min, pH 8.5. Following neutralization, sections were again rinsed in TBS followed by incubation in blocking buffer (10% normal horse serum and 0.3% Triton X-100 in TBS) for 1 h and incubation in sheep anti-BrdU (1:250; catalog #ab1893, Abcam) in blocking buffer overnight at 4°C. The following day, slices were washed in TBS and incubated for 1.5 h in donkey anti-sheep (1:200; Alexa Fluor 594; catalog #ab150184, Abcam) at room temperature. Once labeled, sections were washed in TBS, mounted, and coverslipped using anti-fade containing DAPI (ProLong Gold; catalog #P36931, Molecular Probes).

### Doublecortin labeling

Doublecortin studies were performed using tissue collected from BrdU-injected mice. Tissue sections were washed in a 1× Tris buffer, pH 7.6, prior to incubation in 1% H_2_O_2_ for 30 min to quench endogenous peroxidase activity. Sections were then washed in the 1× Tris buffer, and cells were permeabilized using 0.1% Triton in Tris for 15 min. Sections were then incubated in a solution of 0.05% bovine serum albumin (BSA) and 0.1% Triton in Tris for 15 min prior to blocking in a blocking buffer solution of 0.05% BSA, 10% natural horse serum, and 0.1% Triton X-100 in Tris for 1 h. After blocking, tissue was rinsed in 0.1% Triton X-100 in Tris and 0.1% Triton X-100 with 0.05% BSA in Tris for a period of 15 min each prior to incubation in rabbit anti-doublecortin (1:1000; catalog #AB18723, Abcam) in blocking buffer solution overnight at 4°C. Sections were again rinsed in 0.1% Triton X-100 in Tris and 0.1% Triton X-100 with 0.05% BSA in Tris for a period of 15 min each prior to incubation in a biotinylated anti-rabbit IgG (1:200; catalog #BA-1000, Vector Laboratories) for 2 h at room temperature. Sections were then briefly rinsed in 0.1% Triton X-100 with 0.05% bovine serum albumin in Tris and incubated with avidin–biotin complex (1:1000; ABC Kit, catalog #PK-6100, Vector Laboratories) in the same solution for a period of 1 h. Tissue was then incubated in diaminobenzidene with nickel according to manufacturer instructions (catalog #SK-4100, Vector Laboratories) for ∼2 min and washed four times in 1× Tris. Sections were then mounted and coverslipped.

### Cell counting

Brain slices labeled for BrdU and doublecortin were imaged at 10× magnification using a 1344 × 1024 CCD camera (Orca-ER, Hamamatsu) on a DMI6000B Microscope (Leica). Images were subsequently analyzed off-line using ImageJ (version 1.48, National Institutes of Health). Individual images of the granule cell layer were concatenated, and a region of interest (ROI) consisting of labeled cells within 35 µm of the subgranular zone was selected and straightened. The number of cells positive for BrdU or doublecortin was then counted visually by an experimenter blind to genotype and divided by the ROI, producing a density value. Density values were then normalized to the density of BrdU-positive or doublecortin-positive cells in wild-type mice.

### Dentate gyrus granular cell layer measurements

The overall width of the granular cell body layer of the dentate gyrus was assessed using MATLAB code (modifed from Temme et al., 2015), which was designed to calculate the average width of a region of interest. The collection of sections was standardized across mice, with four slices analyzed per mouse spaced evenly throughout the dentate gyrus, specifically, every 12th slice of 40 μm slices starting from the anterior end. After acquisition, slices were stained for DAPI to identify the cell body layer. Images of each slice was taken at 10× magnification and loaded into a predesigned MATLAB code that identifies the DAPI-labeled cell body layer for the apex, and the superior and inferior blades of the dentate gyrus. Once the cell body layer is identified, the program calculates the width of each blade per micrometer (pixel density) along the entire length of each blade respectively. The program then outputs the average width value per blade. These outputted values are then averaged per dentate gyrus, then per mouse, then per genotype.

### Statistical analysis

The analysis of behavioral experiments was performed using repeated-measures ANOVAs, two-way ANOVAs, and planned unpaired *t* tests comparing Ca_V_1.2^cKO^ mice with their wild-type counterparts. Learning across fear-conditioning discrimination ratio, the standard Morris water maze, the limited cues water maze, and the visible platform test in the limited cues water maze were analyzed using repeated-measures ANOVA, with genotype and training as factors. Discrimination freezing was analyzed using a repeated-measures ANOVA with genotype and context as factors. Platform preference during water maze probe tests was analyzed using a one-sample *t* test was used, with chance (25%) set as the hypothetical mean. The context test and differences in BrdU and doublecortin cell densities were analyzed using an unpaired *t* test.

## Results

### Conditional deletion of Ca_V_1.2 does not affect simple hippocampal-dependent learning

**Figure 1.**
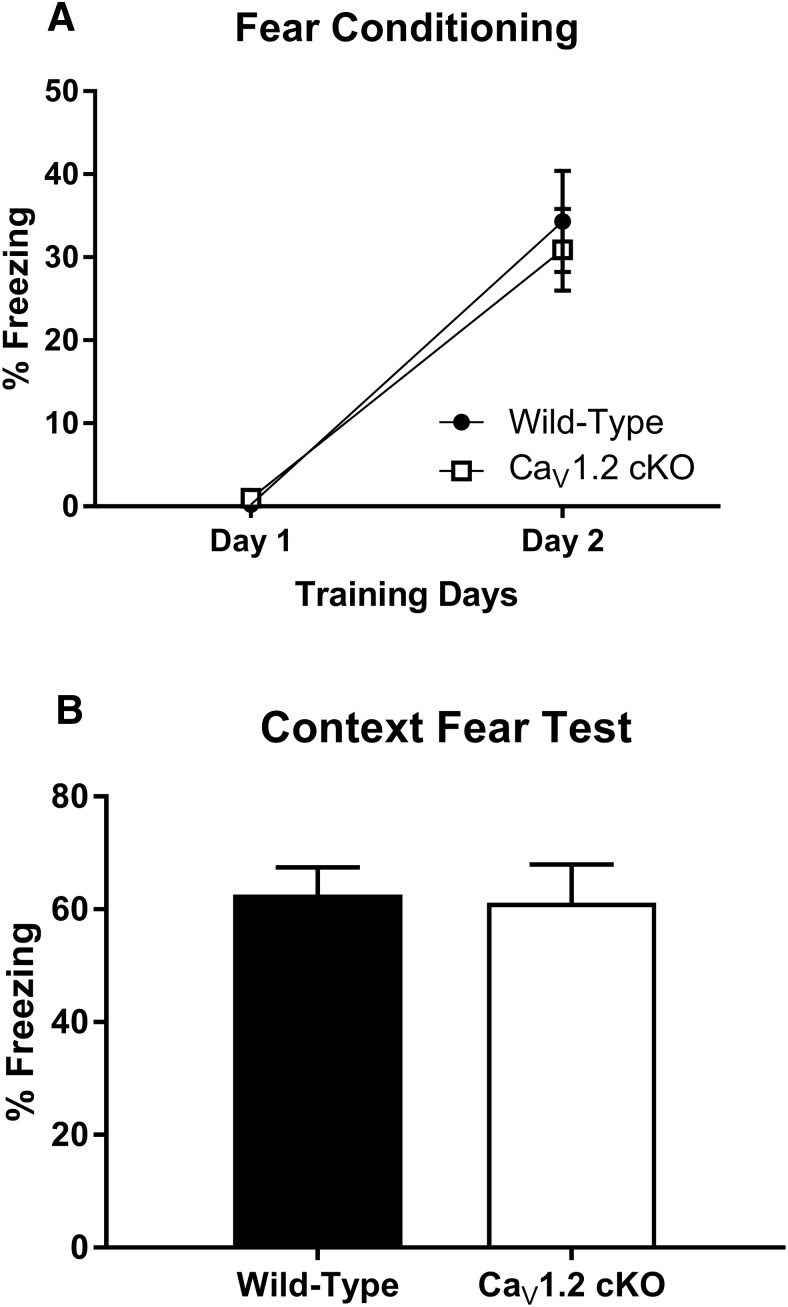
Neuronal deletion of Ca_V_1.2 does not impair fear acquisition or consolidation to a conditioned context. Mice were fear conditioned to a context using three unsignaled footshocks per day for 2 d. ***A***, Both Ca_V_1.2 conditional knock-out mice (*n* = 17) and wild-type mice (*n* = 17) exhibited a significant enhancement in freezing in response to the conditioned context between days 1 and 2 of training. ***B***, When tested for their fear of the context, Ca_V_1.2 conditional knock-out mice exhibited high levels of freezing comparable to those of wild-type mice. Data are represented as the mean ± SEM.

While mice in which the deletion of Ca_V_1.2 is restricted to glutamatergic neurons in the forebrain do not exhibit deficits in either contextual fear conditioning (McKinney et al., 2008) or the Morris water maze 24 h after training (White et al., 2008), it is possible that the deletion of Ca_V_1.2 in neurons throughout the brain could generate deficits in these forms of learning. Using the synapsin1-cre Ca_V_1.2^cKO^ mice, we investigated the role of Ca_V_1.2 in contextual fear conditioning. Mice were fear conditioned to a context across 2 d using one session per day. Each session consisted of 3 min of context exposure followed by three unsignaled footshocks (0.5 mA, 2 s) with an intershock interval of 30 s. The acquisition and consolidation of fear across training days was analyzed by comparing the average percentage of freezing during the 3 min of context exposure for each day (Fig. 1*A*). The analysis of context conditioning using a two-way ANOVA found a significant effect of training day (*F*_(1,32)_ = 66.58, *p* < 0.0001), but not of genotype (*F*_(1,32)_ = 0.12, *p* = 0.73). Both wild-type and Ca_V_1.2^cKO^ mice showed an increase in freezing across training with an average percentage of freezing of 0.2% on day 1 and 34% on day 2 in wild-type mice, and 1% on day 1 and 31% on day 2 in Ca_V_1.2^cKO^ mice. Twenty-four hours after the last training session, mice were tested for their fear of the context using 5 min of context exposure. The analysis of freezing behavior during context testing using an unpaired *t* test found no significant difference between genotypes (*p* = 0.86) with an average percentage of freezing of 63% in wild-type mice and 61% in Ca_V_1.2^cKO^ mice (Fig. 1*B*). These data suggest normal fear conditioning in mice lacking Ca_V_1.2.

**Figure 2.**
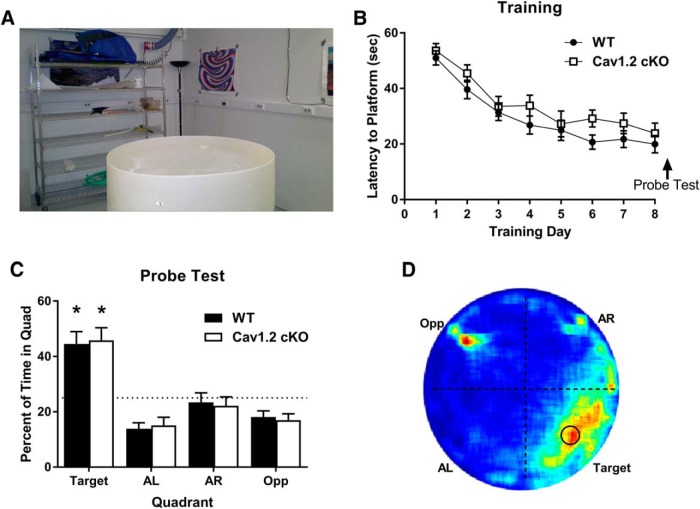
Neuronal deletion of Ca_V_1.2 does not impair spatial learning in the standard Morris water maze. ***A***, Mice were trained to find a hidden platform in a Morris water maze using numerous spatial cues around the room. Mice were trained across 8 d with a probe test on day 9. ***B***, Both Ca_V_1.2 conditional knock-out mice (*n* = 18) and wild-type mice (*n* = 24) exhibited a significant decline in the latency to find the hidden platform across training days. ***C***, When probed for their memory of the platform location, both Ca_V_1.2 conditional knock-out mice and wild-type mice spend significantly more time in the target quadrant than chance [*p < 0.0001, single-group *t* test with a hypothetical mean of 25% (indicated by dashed line)]. ***D***, Representation of the time spent throughout the water maze during the probe test using a heat map illustrated a strong preference for the platform location and the target quadrant, with an additional strong signal at the location at which mice were released into the pool.

To further explore the effects of Ca_V_1.2 on hippocampal-dependent learning, we tested Ca_V_1.2^cKO^ mice in the standard Morris water maze task (Morris, 1984). In the standard Morris water maze, mice learn to use the complex spatial cues located distal to the water maze to locate a hidden platform placed just below the surface of the pool (Fig. 2*A*). Mice were trained to find the location of the hidden platform using two sessions per day for 9 d. During each session, mice were pseudo-randomly released into the pool and allowed 60 s to locate the hidden platform. Approximately 24 h after the last day of training, mice were tested for their spatial memory of the platform location using a probe test. Analysis of the latency to find the hidden platform using a repeated-measures ANOVA exhibited a significant reduction in latency to find the platform across training days (*F*_(8,280)_ = 25.85, *p* < 0.0001) with a decrease in average latency from 50.0 to 23.4 s in wild-type mice and 53.6 to 19.5 s in Ca_V_1.2^cKO^ mice, but no difference between genotypes (*F*_(1,40)_ = 3.147, *p* = 0.09; Fig. 2*B*).

During the probe test, mice were analyzed for the percentage of time spent in the location in which the platform had previously been located. Exploration in the probe test was analyzed by splitting the maze into four quadrants with the original platform location in the center of one quadrant, titled the target quadrant. The percentage of time spent in the target quadrant was then compared to a chance level of 25%. During the probe test, both Ca_V_1.2^cKO^ mice and their wild-type littermates spent a significant percentage of time in the target quadrant compared with chance (*p* < 0.0001 and *p* < 0.0001, respectively, one sided *t* test; Fig. 2*C*). Similarly, the percentage of time that the Ca_V_1.2^cKO^ mice and their wild-type littermates spent in the target quadrant was the same (unpaired *t* test, *p* = 0.844). This is further illustrated using a heat map to illustrate the average percentage of time spent throughout the probe test (Fig. 2*D*). Examination of the heat map revealed a large period of time spent in the target quadrant where the hidden platform was located. A significant amount of time spent in the opposite quadrant was also noted, which corresponded with the location at which mice were released into the pool and likely represents the brief period during which the mice spent orienting themselves. Similar to previously published literature, these data suggest that Ca_V_1.2 is not involved in spatial learning in the standard Morris water maze when assessed 24 h after training (White et al., 2008). Together with the data demonstrating no effects of the deletion of Ca_V_1.2 on fear conditioning to a context, this suggests that Ca_V_1.2 does not affect these forms of learning and memory, which previously were found to be neurogenesis-independent forms of hippocampal-dependent learning tasks (Shors et al., 2002; Jaholkowski et al., 2009).

### Neuronal deletion of Ca_V_1.2 produces deficits in dentate gyrus-associated learning tasks

**Figure 3.**
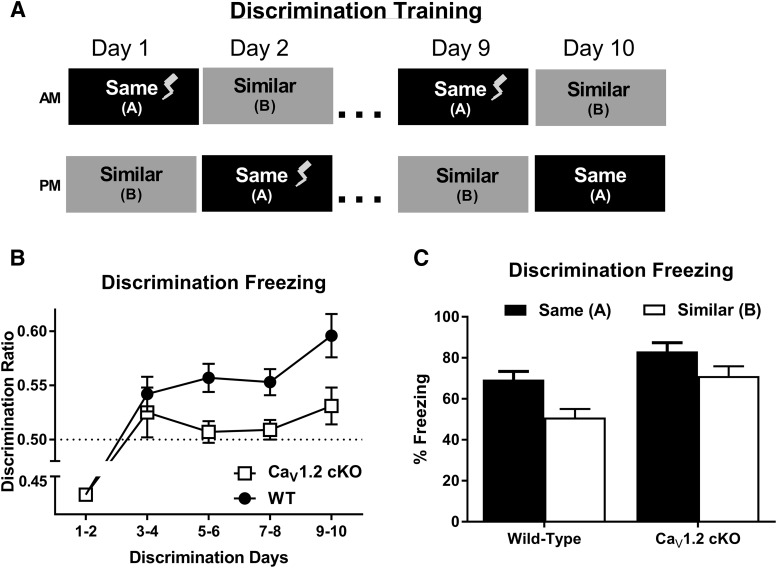
Ca_V_1.2 conditional knock-out mice exhibit significant deficits in context discrimination. ***A***, Mice were trained to discriminate between two similar contexts through context exposure to each context once a day for 10 d with one context, and the same context, paired with a footshock. Context discrimination throughout training was assessed using a discrimination ratio, with a ratio of 0.5 representing a lack of discrimination between the two contexts. ***B***, While wild-type mice (*n* = 18) displayed a significant discrimination ratio >0.5 (indicated by a dashed line) by day 3–4 of training, Ca_V_1.2 conditional knock-out mice (*n* = 17) failed to show a significant discrimination ratio until days 9–10. Additionally, analysis between genotypes revealed a significant deficit in context discrimination in Ca_V_1.2 conditional knock-out mice compared with wild-type mice. ***C***, Comparison of average freezing levels between the trained context, A, and the similar context, B, on days 9–10 of training reveal revealed a significant effect of genotype and context, but no interaction between genotype and context. Data are represented as the mean ± SEM.

To determine whether neuronal deletion of Ca_V_1.2 produces deficits in more difficult versions of hippocampal-dependent learning, Ca_V_1.2^cKO^ mice were tested in the following two alternate versions of contextual and spatial learning: context discrimination and a limited cues version of the Morris water maze. During context discrimination, mice were trained to discriminate between two contexts, contexts A and context B, through exposure to each context once a day for 10 d (Fig. 3*A*). For 9 d, context A, was paired with an unsignaled footshock (0.5 mA, 2 s), and on day 10, mice were placed in context A, but no footshock was given. For all 10 d, mice were placed in context B for the same period of time as context A without a footshock, and the order in which the mice experienced each context was counterbalanced. Discrimination between contexts was calculated as a discrimination ratio, calculated as the average percentage of freezing in the trained context divided by the total percentage of freezing in both contexts per day. Data were analyzed as the average of every 2 d to control for time-of-day effects. Due to the order in which the contexts were presented, mice were analyzed for a freezing response to context A on day 1 prior to the first pairing of the context and footshock, while the first exposure to context B occurred after the pairing of context A with the footshock. Therefore, the context discrimination ratio from days 1–2 do not accurately represent the measurement of discrimination between contexts. Analysis of context discrimination ratios across training days using a two-way repeated-measures ANOVA revealed a significant effect of genotype (*F*_(1,330)_ = 5.183, *p* = 0.0294) with discrimination deficits in Ca_V_1.2^cKO^ mice (Fig. 3*B*). While wild-type mice showed a significant discrimination ratio >0.5 starting on days 3–4 of context discrimination (*p* = 0.0182, one-group *t* test), Ca_V_1.2^cKO^ mice exhibited discrimination ratios that were no better than chance up until days 9–10 of training (*p* = 0.0159, one-group *t* test). Consistent with this observation, the analysis of context discrimination through the comparison of raw freezing levels between the two contexts during the discrimination test using a two-way repeated-measures ANOVA revealed a significant effect of genotype (*F*_(1,33)_ =9.231, *p* = 0.0046) and context (*F*_(1,33)_ = 41.69, *p* < 0.0001), but no significant interaction between genotype and context (Fig. 3*C*). These data suggest a moderate, but significant deficit in context discrimination in mice with a pan-neuronal deletion of Ca_V_1.2, which can be overcome by training.

**Figure 4.**
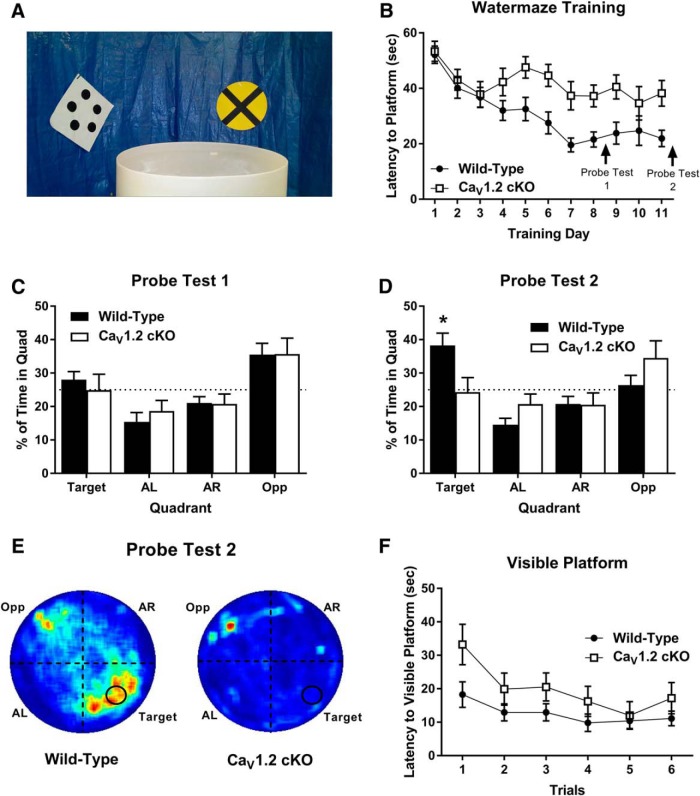
Ca_V_1.2 conditional knock-out mice exhibit significant impairments in the acquisition of spatial memory in the limited cues version of the Morris water maze. ***A***, To test for deficits in complex hippocampal learning, mice were tested in a version of the Morris water maze in which the visible cues around the room were limited. Mice were trained across 11 d and tested for their spatial memory of the platform location on days 9 and 12 of experimentation. ***B***, Across training, Ca_V_1.2 conditional knock-out mice (*n* = 14) exhibited a significant deficit in the latency to find the hidden platform compared with wild-type mice (*n* = 18). ***C***, During the first probe test, neither Ca_V_1.2 conditional knock-out mice nor wild-type mice exhibited a significant preference for the target quadrant compared with chance or the other quadrants. ***D***, During the second probe test, wild-type mice, but not Ca_V_1.2 conditional knock-out mice, exhibited a significant preference for the target quadrant over the other quadrants and over chance [*p < 0.0001, single-group *t* test with a hypothetical mean of 25% (indicated by dashed line)]. ***E***, Heat maps for wild-type mice (left) and Ca_V_1.2 conditional knock-out mice (right) during probe trial 2. ***F***, When tested for their ability to find a visible platform, both Ca_V_1.2 conditional knock-out mice and wild-type mice were able to find the platform across six trials. Data are represented as the mean ± SEM.

To further investigate the effects of neuronal deletion of Ca_V_1.2 in a more difficult spatial learning task, we assessed the performance of Ca_V_1.2^cKO^ mice in a version of the water maze that we refer to as the “limited cues” water maze. During the limited cues water maze, complex spatial cues around the room were eliminated using a blue plastic barrier, and, in their place, four discreet cues were spaced evenly around the maze (Fig. 4*A*). Mice were trained to the limited cues water maze using two 60 s training sessions per day, for 11 d. Training sessions were performed as in the standard Morris water maze. Mice were probed for their preference for the platform location on day 9, similar to the standard water maze, and on day 12, 24 h after the last training session.

Analysis of the latency to find the hidden platform across training using a repeated-measures ANOVA, and genotype and training day as factors, revealed a significant effect of both genotype (*F*_(1.30)_ = 12.362, *p* = 0.0014) and training day (*F*_(13,390)_ = 8.343, *p* < 0.0001) with Ca_V_1.2^cKO^ mice exhibiting a significant deficit in training compared with wild-type mice (Fig. 4*B*). While wild-type mice showed a decrease in latency to find the hidden platform from 52.097 s on day 1 to 21.923 s on day 11, Ca_V_1.2^cKO^ mice only showed a minor improvement in latency to find the hidden platform from 53.348 s on day 1 to 38.207 s on day 11. During probe test 1, on day 9 of training, neither wild-type nor Ca_V_1.2^cKO^ mice showed a significant preference for the target quadrant, represented as a percentage of time, compared with chance (*p* = 0.23 and *p* = 0.98, respectively; one group *t* test; Fig. 4*C*). These data demonstrate that, after 8 d of training, neither Ca_V_1.2^cKO^ mice nor wild-type mice have a spatial memory for the platform location, and suggest that this version of the water maze is indeed more difficult to learn. During probe test 2, on day 12 of experimentation, wild-type mice exhibited a significant preference for the target quadrant compared with chance (*p* = 0.0021; Fig. 4*D*,*E***)**. However, Ca_V_1.2^cKO^ mice did not spend significantly more time searching in the target quadrant compared with chance (*p* = 0.86). Furthermore, an unpaired *t* test comparing the percentage of time spent in the target quadrant revealed a difference, with the wild-type mice spending significantly more time in the quadrant where the platform was previously located (*p* = 0.0189). These data suggest that wild-type mice, but not Ca_V_1.2^cKO^ mice, formed a spatial memory of the platform location. Following probe test 2, Ca_V_1.2^cKO^ mice and wild-type mice were tested for their ability to find a visible platform across six trials (Fig. 4*F*). Poor performance on the visible platform could suggest an inability for mice to properly view the spatial cues of the maze. Comparison of the latency to find the visible platform between genotypes using a repeated-measures ANOVA revealed no significant difference in latency to find the platform between genotypes (*F*_(1,30)_ = 2.424, *p* = 0.1300) but a significant effect of training trials (*F*_(5,150)_ = 3.010, *p* = 0.0128), suggesting that deficits in spatial learning in Ca_V_1.2^cKO^ mice are not likely due to a deficit in their ability to see the visible cues or their ability to perform the task. Together, these data suggest that neuronal deletion of Ca_V_1.2 produces deficits in difficult hippocampal tasks, such as those associated with dentate gyrus neurogenesis (Shors et al., 2002; Saxe et al., 2006).

### Neuronal deletion of Ca_V_1.2 impairs neurogenesis and cell division in the dentate gyrus

**Figure 5.**
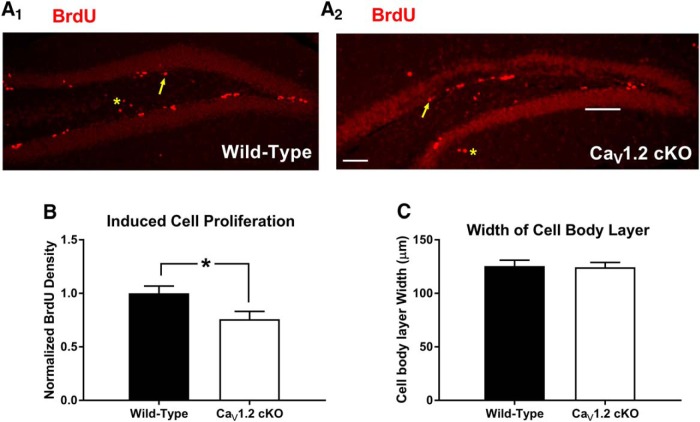
Ca_V_1.2 conditional knock-out mice exhibit decreased levels of cell division in the adult dentate gyrus. ***A_1_***, ***A_2_***, Ca_V_1.2 conditional knock-out mice (*n* = 10) and wild-type mice (*n* = 7) were also assessed for the rates of cell division in the adult dentate gyrus. Arrows indicate examples of BrdU-positive cells within the area of interest. Asterisks represent examples of cells outside of the area of interest. ***B***, Comparison of the density of BrdU-positive cells in the dentate gyrus between genotypes revealed a significant decrease in cell division in Ca_V_1.2 conditional knock-out mice vs wild-type mice. ***C***, Ca_V_1.2 conditional knock-out mice did not exhibit alterations in the width of the dentate gyrus. Data are represented as the mean ± SEM. *p < 0.05, unpaired *t* test.

Previous experiments using pharmacological methods have implicated LVGCCs in cell proliferation and neurogenesis prenatally (D'Ascenzo et al., 2006; Piacentini et al., 2008; Brustein et al., 2013) and into adulthood (Deisseroth et al., 2004; Luo et al., 2005; Zhu et al., 2012). In light of previous literature suggesting that context discrimination and difficult hippocampal tasks in general can be influenced by adult neurogenesis in the dentate gyrus (Saxe et al., 2006; Sahay et al., 2011) and the abundance of Ca_V_1.2 expression in the dentate (Hell et al., 1993), we hypothesized that the deficits we observed in the Ca_V_1.2^cKO^ mice might reflect changes in cell proliferation and neurogenesis in the dentate gyrus. To determine whether neuronal deletion of Ca_V_1.2 alters cell proliferation and adult neurogenesis, we labeled dividing cells in the dentate gyrus in naive mice using 5 d of 100 mg/kg BrdU (del Rio and Soriano, 1989) injections administered intraperitoneally. Twenty-four hours after the final BrdU injection, mice were perfused, and sections containing the subgranular zone of the dentate gyrus were collected and processed for BrdU using a red fluorescent secondary antibody (Fig. 5*A1*,*A2* ). Cells that were BrdU positive were counted and normalized to the average density of BrdU in wild-type mice. Cell counts revealed that pan-neuronal deletion significantly reduced the number of BrdU-positive cells in Ca_V_1.2^cKO^ mice compared with wild-type mice (*p* = 0.04, unpaired *t* test) with a decrease in BrdU-positive cell density of 24.2% in Ca_V_1.2^cKO^ mice (Fig. 5*B*). Analysis of the width of the cell body layer of the dentate gyrus (Fig. 5*C*) using an unpaired *t* test revealed no significant difference between genotypes (*p* = 0.8782). These data suggest that Ca_V_1.2 plays an important role in cell proliferation in the adult dentate gyrus.

**Figure 6.**
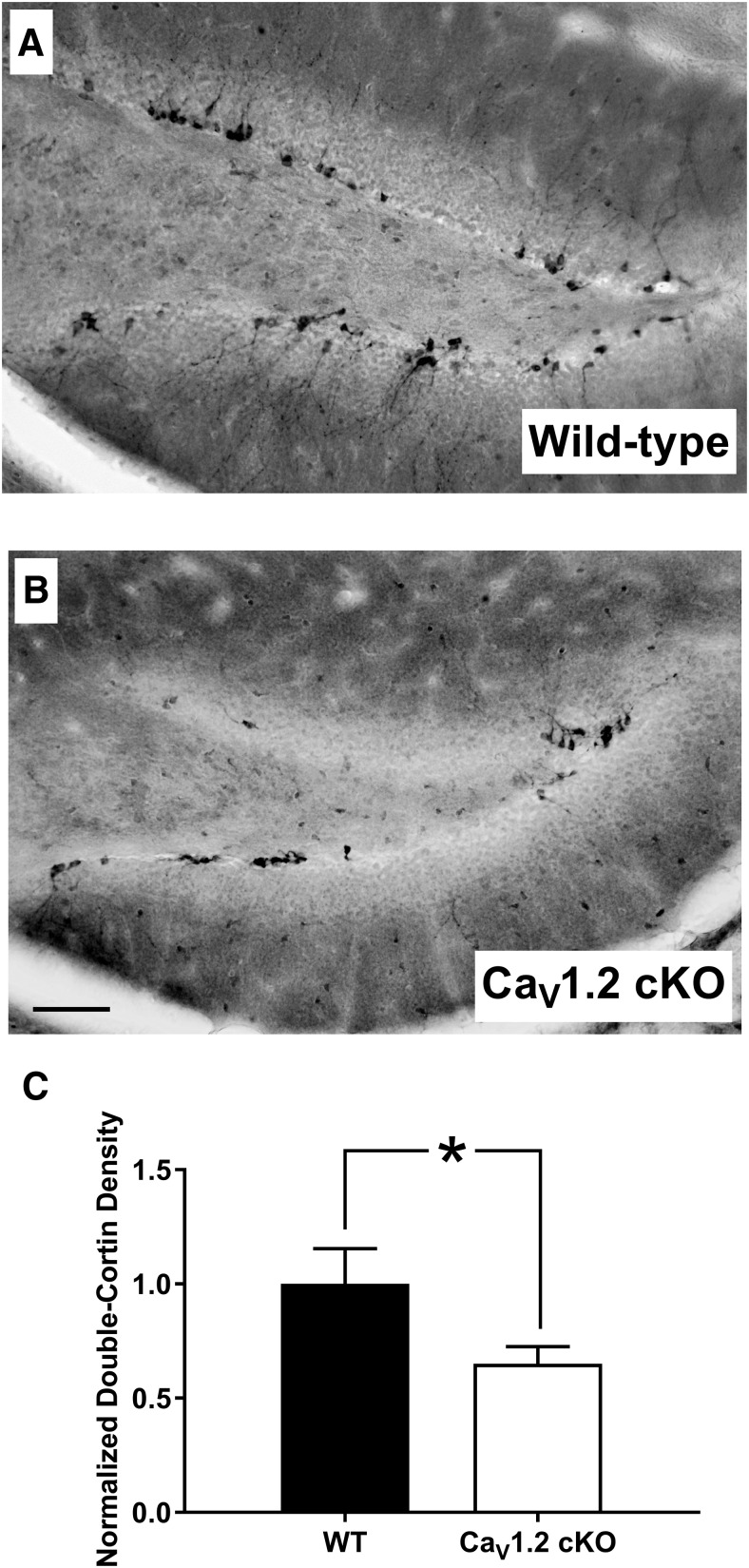
Ca_V_1.2 conditional knock-out mice exhibit decreased levels of immature neurons in the adult dentate gyrus. ***A***, ***B***, Both Ca_V_1.2 conditional knock-out mice (*n* = 10) and wild-type mice (*n* = 7) were assessed for adult-born immature neurons in the dentate gyrus through immunohistological labeling of doublecortin-positive cells in the subgranular zone of the dentate gyrus. ***C***, Analysis of the density of doublecortin-positive cells revealed a significant decrease in adult-born immature neurons in Ca_V_1.2 conditional knock-out mice compared with wild-type mice. Data are represented as the mean ± SEM. *p < 0.05, unpaired *t* test.

A recent report suggested that there is strong expression of Ca_V_1.2 in immature doublecortin-positive cells within the dentate gyrus (Marschallinger et al., 2015). Therefore, we wanted to determine whether the deletion of Ca_V_1.2 could alter the number of newborn neurons within the dentate gyrus. To assess the number of newborn neurons within the dentate gyrus, we performed immunohistochemistry for doublecortin (Brown et al., 2003; Fig. 6*A*). Similar to BrdU studies, doublecortin analysis was limited to the subgranular zone of the dentate gyrus. Analysis of the number of immature neurons in the dentate gyrus using doublecortin staining in the dentate gyrus revealed a significant decrease in the density of doublecortin-labeled cells (*p* = 0.04), with Ca_V_1.2^cKO^ mice exhibiting a decrease in doublecortin-labeled cells of 34.7% compared with wild-type mice (Fig. 6*B*). Although it remains unclear whether Ca_V_1.2 alters the number of immature neurons via a role in differentiation, early neuronal survival, or cell proliferation, our data suggest that pan-neuronal deletion of Ca_V_1.2 decreases the number of newborn neurons in the dentate gyrus. Together, the deletion of Ca_V_1.2 alters the number of newborn neurons and the rate of cell proliferation in the dentate gyrus, which could result in deficits in hippocampal-dependent learning, such as those seen in Ca_V_1.2^cKO^ mice.

## Discussion

Using transgenic mice with a pan-neuronal deletion of the LVGCC Ca_V_1.2, we investigated the role of Ca_V_1.2 in hippocampal-dependent learning and cognition, with a focus on simple versus complex tasks. Because of the implication of the dentate gyrus in cognitively demanding tasks along with previous literature suggesting a role of LVGCCs in neural differentiation, and the abundance of Ca_V_1.2 expression in the dentate gyrus compared with other parts of the hippocampus, we also examined the effects of the deletion of Ca_V_1.2 on cell proliferation and neurogenesis in the dentate gyrus.

We tested Ca_V_1.2^cKO^ mice in two types of simple hippocampal-dependent learning tasks: Pavlovian fear conditioning and the standard Morris water maze. Ca_V_1.2^cKO^ mice exhibited normal acquisition, consolidation, and expression of context-based fear memories. Additionally, Ca_V_1.2^cKO^ mice acquired a spatial memory of the location of a hidden platform in the standard Morris water maze at rates and levels that were comparable to those observed in wild-type mice. However, when tested in the context discrimination task, Ca_V_1.2^cKO^ mice exhibited a significant deficit in the ability to discriminate two contexts throughout training. When tested in the limited cues water maze, in which the number and diversity of spatial cues are limited compared with the standard water maze, Ca_V_1.2^cKO^ mice exhibited significant deficits in the latency to find the hidden platform compared with wild-type mice and failed to show a preference for the target quadrant when probed for their memory for the platform location.

Previous studies investigating the role of Ca_V_1.2 in hippocampal-dependent learning have yielded conflicting results. While the deletion of Ca_V_1.2 in excitatory neurons did not alter behavioral performances in the Morris water maze and contextual fear conditioning in one set of article (McKinney et al., 2008; White et al., 2008), it did produce deficits in a version of the Morris Water maze termed the water maze spatial-discrimination task and the labyrinth maze in another (Moosmang et al., 2005). Though these conflicting results could be attributed to differences in the transgenic models used, our studies suggest that both sets of data may be valid, with one set supporting the simple hippocampal tasks and behavioral results found in our mice with neuronal deletions of Ca_V_1.2, while the other set of results may reflect the role of Ca_V_1.2 in more complex versions of hippocampal learning. In the water maze spatial-discrimination task, mice must learn to discriminate the spatial location of two visible platforms, one that is fixed and another that sinks (Arns et al., 1999; Steckler et al., 1999; Kleppisch et al., 2003; Moosmang et al., 2005). In the labyrinth maze, mice learned to transverse a brightly lit maze in order to be returned to their shadowed home cages. The maze was made up of nine intersections at which point mice must make a spatial decision in direction (Tang et al., 1999; Moosmang et al., 2005). Though the exact difficulty of these tasks compared with other hippocampal-dependent tasks is hard to assess, impairments of Ca_V_1.2 in difficult but not simple hippocampal learning tasks may explain the discrepancy in results found in previous articles.

In previous studies, our selected simple hippocampal-dependent learning tasks have been found to be dependent on the hippocampus proper, but independent of neurogenesis in the dentate gyrus. Previous studies investigating the role of neurogenesis within the dentate gyrus in learning and memory have suggested that decreased neurogenesis has no effect on contextual fear conditioning or the standard Morris water maze (Shors et al., 2002; Jaholkowski et al., 2009). In addition, we defined our difficult hippocampal tasks as similar tasks, which involved similar brain structures, but were more cognitively challenging and therefore required additional training in order to learn. Studies suggest that hippocampal-dependent tasks that are more difficult are more likely to require the dentate gyrus and adult-born neurons within this structure (Shors et al., 2002). This appears to be the case for context discrimination in which decreases in neurogenesis impair the ability of an animal to learn to discriminate between two contexts (Saxe et al., 2006), while increases in neurogenesis enhance the performance of an animal in this task (Nakashiba et al., 2012). Although there is no direct literature investigating the effects of adult neurogenesis on the limited cues water maze, we believe that the limited cues water maze is more difficult than the standard water maze. We found that, while wild-type mice were able to learn the location of a hidden platform in the standard Morris water maze after only 8 d of training, wild-type mice trained in the limited cues version of the water maze exhibited chance performance after 8 d of training and required an additional 3 d of training (six additional training sessions) to learn the location of the platform.

In light of reports linking difficult hippocampal-dependent learning tasks and the birth of new neurons in the dentate gyrus (Shors et al., 2002), in particular the strong link drawn between context discrimination and neurogenesis (Saxe et al., 2006; Nakashiba et al., 2012), we examined the putative role of Ca_V_1.2 in cell proliferation and adult neurogenesis in the dentate gyrus. We found that in naive mice cell proliferation in the dentate gyrus (assessed using BrdU) was reduced, as was the number of doublecortin-positive cells. We believe that these decreases in cell proliferation and immature neurons in Ca_V_1.2^cKO^ mice could produce deficits in hippocampal learning that are dependent on the dentate gyrus, such as in context discrimination and the limited cues water maze. Using pharmacological blockade, studies have linked LVGCCs with cell proliferation and neurogenesis prenatally (D'Ascenzo et al., 2006; Piacentini et al., 2008; Brustein et al., 2013) and into adulthood (Deisseroth et al., 2004; Luo et al., 2005; Zhu et al., 2012). In the case of adult neurogenesis, studies found that the blockade of LVGCCs did prevent induced cell proliferation (Zhu et al., 2012) and neurogenesis (Deisseroth et al., 2004; Luo et al., 2005). These results are furthered by a recent study demonstrating that a loss of Ca_V_1.2 decreased the survival rate of adult-born neurons in the dentate gyrus (Kang et al., 2015; Lee et al., 2016). Additionally, the activation of LVGCCs using channel agonists increased the percentage of neural progenitor cells that survived to become adult neurons (Deisseroth et al., 2004). While previous studies have found large levels of Ca_V_1.2 expression in the adult dentate gyrus, Ca_V_1.2 has not been found in neural stem cells (Deisseroth et al., 2004; Marschallinger et al., 2015). This suggests that Ca_V_1.2 may be altering cell proliferation via its effects on neuronal activity within the dentate gyrus associated with cell proliferation. Based on these results, Ca_V_1.2 is capable of modulating cell proliferation in the adult dentate gyrus and does so in a cell-specific manner. Previous studies have found Ca_V_1.2 to be expressed in doublecortin-positive cells in the dentate gyrus. (Marschallinger et al., 2015). Therefore, Ca_V_1.2 could also be altering the number of immature neurons via the loss of Ca_V_1.2 in the immature neurons themselves. These results are similar to previous studies in which the deletion of Ca_V_1.3 resulted in a decrease in doublecortin-positive and the number of BrdU-positive cells after 4 weeks (Marschallinger et al., 2015). Therefore, both Ca_V_1.2 and Ca_V_1.3 appear to be involved in adult neurogenesis.

Interestingly, these results were seen in the absence of a change in the average width of the dentate gyrus, which may suggest normal dentate gyrus neurogenesis and formation in the absence of Ca_V_1.2 early in development, despite the decrease in adult neurogenesis in Ca_V_1.2^cKO^ mice. Potentially, this could be due to the limited expression of Ca_V_1.2 prenatally compared with postnatally or the influence of neuronal circuitry on adult neurogenesis that is not present prenatally. Despite these implications, we cannot rule out the potential effects of a loss of Ca_V_1.2 on prenatal neurogenesis or the postnatal development of the dentate gyrus based on our results alone. First, while steps were taken to measure the width of the dentate gyrus across the entire length of the structure, it is possible that alterations in neuron number could have been missed or occurred in a way that did not alter the actual width of the structure. In fact, it could be an interaction of alterations in adult and perinatal neurogenesis in the absence of Ca_V_1.2 that could be producing the behavioral deficits. In addition, it is possible that the methods in which mice were treated prior to tissue collection could have impacted dentate gyrus width or cell proliferation detected by BrdU and doublecortin. For example, the use of anesthetics has been linked with alterations in brain physiology and adult neurogenesis (Stratmann et al., 2009; Zhu et al., 2010; Erasso et al., 2013), although the use of anesthetics in our experiments was limited to 30 s of isoflurane exposure on each occasion, which is substantially different than the 3–8 h of aerosolized anesthetic found to alter neurophysiology in previous studies. Additionally, all environmental and temporal features of the experiments performed, particularly regarding the investigation of dentate gyrus structure, were tightly controlled to eliminate variability between mice and groups as much as possible. Finally, we must concede the unlikely possibility that, because our cell-counting experiments were not strictly adherent to unbiased stereology principles, it is possible that the observed differences in the Ca_V_1.2^cKO^ mice might be due to genotype-specific changes in the anatomical structure of the dentate gyrus.

The data presented here provide strong evidence that Ca_V_1.2 does in fact play a role in hippocampal-dependent learning. However, Ca_V_1.2 is required only for tasks that are more cognitively demanding. This may reflect the differential expression pattern of Ca_V_1.2 within the hippocampus. Additionally, we have demonstrated an important role of Ca_V_1.2 in modulating cell proliferation and adult neurogenesis in the hippocampal structure, the dentate gyrus, with this modulation as a candidate mechanism by which Ca_V_1.2 mediates complex, but not simple, hippocampal-dependent learning.

Interestingly, a number of genome-wide association studies have linked single nucleotide polymorphisms (SNPs) in *CACNA1C* to several psychiatric disorders (Sklar et al., 2008), and it has been variously suggested that these SNPs are putative neuropsychiatric risk alleles (Bhat et al., 2012). Although the majority of studies have implicated these risk alleles in bipolar disorder, a number of studies suggest that variations in *CACNA1C* are associated with major depression, schizophrenia, and autism (Green et al., 2010; Nyegaard et al., 2010; Li et al., 2015). Furthermore, there is mounting evidence that *CACNA1C* risk alleles may be associated with deficits in the processing of emotional information (Soeiro-de-Souza et al., 2012; Nieratschker et al., 2015). This, coupled with the emerging idea that adult neurogenesis may contribute to psychiatric disorders, including schizophrenia (Kang et al., 2015), leads us to speculate that a disruption in Ca_V_1.2 function or expression may play a significant role not only in cognition, but also in neuropsychiatric disorders by altering adult neurogenesis and degrading dentate gyrus function.

## Synthesis

Decisions are customarily a result of the Reviewing Editor and the peer reviewers coming together and discussing their recommendations until a consensus is reached. When revisions are invited, a fact-based synthesis statement explaining their decision and outlining what is needed to prepare a revision will be listed below. The following reviewer(s) agreed to reveal their identity: Heather Cameron, Michael Drew.

Summary

This study examines the effects of a neuron-specific conditional deletion of CACNA1C (Cav1.2), achieved via the Synapsin-Cre mice, on spatial/contextual learning and adult neurogenesis. The novelty of this study arises from the use of behavioral tasks that vary in difficulty. The main result is that Cav1.2cKO mice perform normally on standard versions of context fear conditioning (CFC) and the Morris water maze (MWM) but display deficits on versions of these tasks that putatively challenge pattern separation/completion processes attributed to the DG-CA3 network. The behavioral phenotype in the Cav1.2cKO mice is intriguing and will be of interest to people studying DG and adult neurogenesis as it supports the idea that damage to the DG-CA3 network causes rather specific deficits in pattern completion and separation. In general the data were well presented and the manuscript clearly written, however there are numerous typos that should be detected by a careful proofreading.

There are four main points the authors should address in their revised manuscript.

1.The primary concerns of both reviewers related to the choice and description of the methods, particularly in the approach used to estimate DG volume.

Reviewer #1 states: The lack of effect of Cav1.2KO on granule cell layer thickness seems to argue against a global effect on the granule cell population. However, the analysis may not have been sensitive enough to detect an effect. It is unclear how many sections were used for the granule cell layer thickness measurement. There are readily available methods for measuring the volumes of brain structures, which could better address this question.

While Reviewer #2 adds: More information should be provided about the DG volume estimation. The authors state that a "modified Matlab code" was used but provide no information about the purpose of the code or its calculations, inputs, and outputs. There are several standard, validated methods for estimating the volume of a brain structure (see Howard &amp; Reed "Unbiased Stereology"). Ideally, one of these methods should be used. Given that the KO affects neurogenesis, it's surprising not to see some effect on volume.

This concerns should be addressed prior to resubmission.

2.A second major concern was raised by Reviewer #2 regarding the statistics, stating:

The statistical interpretations are inappropriate in a couple places. For the context discrimination experiment (Fig. 3C) unpaired t-tests are used to compare A v B freezing within each genotype. Unpaired t-tests are not appropriate because the comparisons are within-subject. Furthermore, the conclusion that the A v B difference is bigger in WT mice than KO mice is also inappropriate because this conclusion requires an interaction effect and none is reported. The data should be analyzed using a 2 x 2 ANOVA. A similar criticism applies to the analysis of the MWM probe data. Here, RM-ANOVAs are performed within each genotype on the quadrant data. RM-ANOVAs are probably inappropriate because the repeated measures (percent time in each of the different quadrants) are not independent. Because the percents necessarily add up to 100, they are by definition dependent, which is a problem for RM-ANOVA. Moreover, because the genotypes are not explicitly compared in this analysis, one cannot conclude from this analysis that one genotype performed better than the other. The data themselves look quite strong, and I expect the results would hold up under a more rigorous analysis

These issues should also be addressed prior to resubmission.

3.An important point not clear in the methods was raised by Reviewer #2.

The timeline of the behavioral experiments is unclear. Were the same mice used for all experiments? If so, when were the experiments performed? What order? And how much time between experiments? If the experiments were performed in the same mice, this is somewhat problematic because it introduces a confound. Did the impairments arise because of differences in the nature of the assays or because of the order of the assays? In the realm of DG-dependent learning order is potentially important. Several studies suggest that tasks involving reversals or interference are especially sensitive to DG manipulations (Bannerman et al., 2012; Burghardt et al., 2012; Swan et al., 2014; Engin et al., 2015). The use of different versions of the MWM and/or CFC in sequence potentially creates interference. If so, the authors may have obtained a deficit in the KO mice in the second task even if the order of the tasks were reversed.

This concerns should be addressed prior to resubmission.

4.A final point to be addressed in the revised introduction/discussion was raised by Reviewer #1.

Reviewer #1 states: Although the authors are appropriately cautious, they seem to interpret their results as suggesting that the observed decrease in adult neurogenesis is responsible for the behavioral effects seen in the Cav1.2KO mice. Although this is possible, I think that a related explanation is just as likely. Since the deletion is not limited to adulthood, the effect on neurogenesis observed in adulthood likely also occurred in granule cells during development. The behavioral deficits, then, may reflect impairment of the entire granule cell population, i.e., the dentate gyrus, rather than adult neurogenesis specifically. This could also explain how a rather small (roughly 30%) decrease in neurogenesis is associated with a detectable change in behavior - something that is difficult to find in the adult neurogenesis literature (though this could be a matter of the specific tests chosen).

There are additional more minor points that should be easily addressed:

•Was there a male/female difference? The methods state that both sexes were used but not that equal numbers of each genotype were used in each test, and the Results do not report whether there were differences or not.

•Were mice age-matched across genotype for behavior experiments? For BrdU experiments? How old were the mice for BrdU experiments? Rates of neurogenesis are highly sensitive to age, and 3-7 months old is a wide range.

•What was the concentration of BrdU in solution and was the pH neutralized by adding NaOH to the solution as is commonly done? Why were the mice anesthetized prior to BrdU injection? This is uncommon and will likely decrease cell proliferation due to stress, possibly minimizing the difference due to genotype. It is not clear whether BrdU labeled cells were counted in matched sections or how many sections.

•More information should also be provided about the data collection in the MWM. E.g., what tracking system was used, what data were collected? What was the intertrial interval in the MWM?

•P. 20: the description of Moosmang 2005 is confusing. This study is said to use the visible platform MWM, but presumably it was a different version of the visible platform (discrimination task) than what was used here. If so, then the Moosmang task should be called something else.

•Is anything known about the developmental patterns of CACNA1C expression? When does expression turn on and where? Many genes that are expressed very specifically in the adult are expressed more broadly in the embryo. The possibility that the current results reflect developmental effects should be addressed.
